# Supplementary material for: Are responders to patient health surveys representative of those invited to participate? An analysis of the Patient-Reported Outcome Measures Pilot from the Australian Orthopaedic Association National Joint Replacement Registry
Source: PLoS One. 2021 Jul 2;16(7):e0254196. doi: 10.1371/journal.pone.0254196 (PMC8253407; doi:10.1371/journal.pone.0254196)
Supplement: Attachment — Submitted filename: Response to reviewers_v5.docx [file pone.0254196.s001.docx]

14 April 2021

Mathieu F. Janssen, Ph.D.
Academic Editor
PLOS ONE

Re: Manuscript PONE-D-20-33705: Are responders to patient health surveys representative of those invited to participate? An analysis of the Patient-Reported Outcome Measures Pilot from the Australian Orthopaedic Association National Joint Replacement Registry.

Thank you for reviewing our manuscript. We agree with the points raised and consider the manuscript to be significantly improved. Responses to editorial and reviewer comments are provided below, under each individual italicized, bolded comment. Any changes to the manuscript are provided below each response, indented and a copy of the tracked changes version of the manuscript will be submitted separately.

**Editorial comments**

***It should be made more clear what the flow of included numbers of patients throughout the analysis is: why were respondents excluded from the total of 18.080 to 11.988 (apart from the 1600 mentioned)***

This has been addressed and clarified in the results section. The 18,080 included patients who were later excluded (approximately 5,000 were revisions, not OA, or landline only). A further group had their data externally uploaded from a separate source (another exclusion). However, the original demographic analysis (Tables 1 and 2) included all patients used in the pilot program, whereas analyses including telephone follow up (later tables) were restricted to the time period when telephone follow up was used (this ceased in January 2020). To simplify the paper, we have now restricted ALL analyses to the time period when telephone follow up was used.

The Methods have been edited as follows:

“Between 30 July 2018 and 28 January 2020, the AOANJRR conducted the first stage of a PROMs pilot study, collecting PROMs data from patients pre-operatively and at six months post-surgery from 43 institutions across Australia, including metropolitan and regional, and private and public hospitals from all states and one territory. The analysis includes procedures registered (pre-op analysis) or performed (post-op analysis) between 30 July 2018 and 29 May 2019, to allow 8 months for follow up.”

The results section has been edited as follows:

“Over the study period, 6,224 patients at 43 participating institutions having electronic contact information and primary THA, TKA or TSA for osteoarthritis were registered into the PROMs pilot study.

Of the 6,224 patients registered into the PROMs system, 417 patients had their pre-operative PROMs data collected directly by separate hospital systems, external to the electronic data capture system, and were excluded from pre-op analyses. A further 48 patients were excluded from pre-op analyses due to pre-op follow up phone calls ceasing. For post-op analyses, of the 6,224 patients registered, 24 died and 55 opted out prior to completing their post-op PROMs and were excluded from post-op analyses. A further 573 patients had their procedure after 29 May 2019 and were unable to be followed up by telephone due to post-op follow up phone calls ceasing and are excluded from post-op analyses.

For pre-op analyses, a total of 5,759 patients with a mobile phone and/or email address listed were matched to 6,095 primary hip, knee or shoulder replacement procedures performed for osteoarthritis over the same period and are included in the pre-op analyses. For post-op analyses, a total of 5,572 patients were matched to 5,892 primary hip, knee or shoulder replacement procedures performed for osteoarthritis and are included in the post-op analyses. For patients with multiple procedures, only the first procedure was included in the demographic analyses.”

***It should also be made clear why fewer than 50% of the THA and TKA patients completed PROMs (differences between tables 1 and 2; and tables 3 and 4). And, more importantly, make sure to interpret your findings in light of any dropout that was not clearly reported, and discuss your results and interpretations critically in light of the small rates of available PROM data (in tables 3 and 4).***

Table 2 shows the total population whereas Tables 3 and 4 are broken down into hip and knee, respectively. Furthermore, Table 2 previously included patients recruited after telephone follow up had completed. To simplify the flow of patients, the analyses have now been restricted to patients who were recruited when telephone follow up was being used. The numbers in the tables now align. See response above.

***Discuss why others socio-demographics were not included (namely, education, income, ethnicity), or I would recommend to include these in your analysis.***

These sociodemographic variables are not collected by the Registry and so were not available for analysis. This has been clarified by adding a sentence to the methods:

“Other demographic information (e.g., education and ethnicity) was not available for analysis.”

***I recommend to further investigate methods to answer research question 2 (improving representativeness), or further explain and argue why the current analyses allows you to answer this research question.***

We have added a new table (Table 3) which allows direct comparison of the representativeness of electronic-only follow and electronic + telephone follow up. The following paragraph has been added to the Results:

“Characteristics of electronic-only responders, all responders (electronic and telephone) and all patients are provided in Table 3 to quantify the difference in representativeness when telephone responders are added to electronic-only responders (compared to the total group). Statistical tests are not provided as nearly all differences were statistically significant due to the large sample size. There is no more than 1% or one unit (for age and BMI) difference between electronic-only and all responders (electronic plus telephone), except for post-operative ASA class where the addition of telephone follow up increased the response of ASA class 3 patients.”

***Also, interpret your findings in light of the small initial differences found between responders and non-responders. Differences between responders and non-responders are small, but some are significant. As these are due to sample size, the notion of clinically meaningful differences versus statistical significant differences need to be discussed.***

We now acknowledge this point in the discussion with the addition of the following sentence:

“The small differences seen may not be clinically important and statistical significance, where present, likely reflects the large sample size and corresponding statistical power.”

***Consider to rephrase or re-address research question 3, as the two groups are not only different in terms of mode of administration but because they did or did not respond initially/electronically. Clearly try to distinguish throughout the manuscript between mode of administration (electronic vs telephone) and the value of follow-up in itself.***

We have addressed this specific point in our response to Reviewer 1, below. We have rephrased research question 3 as follows:

“are patient-reported outcomes different between those who provide direct electronic data entry and those who provide information via telephone call after not responding electronically?”

We have also clarified this by adding the following sentence to the discussion:

“It should be noted that these findings do not allow a direct comparison of primary telephone and electronic follow up, as the telephone follow up was only used for patients who did not or could not respond electronically. Rather, it compares electronic follow up to telephone follow up in people who did not respond electronically.”

***Finally, looking at (average) within person differences pre-and post-operation related to responder bias would be a useful addition to the study.***

Unfortunately, we cannot answer the question about the association between response rate and changes in PROMs because non-responders have not provided PROMs data. We have conducted a separate analysis to answer a separate research question (what is the association between pre-operative PROMs and post-operative response rate?). We feel that adding that analysis to this manuscript would not address the main question of this paper (representativeness) and we aim to publish that analysis separately.

We would be able to publish differences in PROMs between the electronic and telephone responders in this manuscript, but feel that it is not directly related to the research question regarding representativeness based on patient characteristics rather than patient responses.

**Reviewer 1**

***The abstract should state the response rates with and without telephone follow-up. This is key information to allow readers to interpret the results reported here.***

The following sentence has been added to the Results section of the Abstract.

“Pre-operatively, 73.2% of patients responded electronically and telephone follow-up of non-responders increased this to 91.4%.”

***The analysis presented in this paper does not answer research question 2, i.e. whether “adding telephone call follow-up to those responding electronically improves representativeness”. The analyses presented in Tables 1 and 2 do not permit quantifying improvements in representativeness e.g. as a % reduction in bias. Instead, the authors present two separate analyses, both of which show a mismatch between responders (however defined) and non-responders. The reader is left to wonder whether there are improvements in representativeness as a result of adding telephone follow-ups. The statistical literature on matching and standardised mean differences may offer some clues on how to formally test for improvement in representativeness as a result of adding a telephone follow-up. It is also worth noting that there does not appear to be much imbalance between electronic responders and non-responders in the first place and, therefore, there may only be limited scope to improve representativeness?***

We agree with these comments. We have added a new table (Table 3) which allows direct comparison of the representativeness of electronic-only follow and electronic + telephone follow up (with the ability to compare these to each other, and the total group). The following paragraph has been added to the Results:

“Characteristics of electronic-only responders, all responders (electronic and telephone) and all patients are provided in Table 3 to quantify the difference in representativeness when telephone responders are added to electronic-only responders (compared to the total group). Statistical tests are not provided as nearly all differences were statistically significant due to the large sample size. There is no more than 1% or one unit (for age and BMI) difference between electronic-only and all responders (electronic plus telephone), except for post-operative ASA class where the addition of telephone follow up increased the response of ASA class 3 patients.”

***The phrasing of research question 3 is potentially misleading. As I understand it, the ‘telephone’ group in Tables 3a-4b includes individuals who failed to report data electronically, i.e. telephone follow-up is conditional on non-participation in electronic data collection. The research question 3 (“are patient-reported outcomes different between those who provide information via telephone call and those who provide direct electronic data entry?”) could be misread as comparing two modes of administration in the same population group, for example, to inform clinical registries on whether to design a PROM programme around electronic or telephone mode of data collection. The data in this study cannot answer this more general question since the two populations are systematically different. The telephone group reflects individuals who do not (wish to) take part in electronic data collection, i.e. a conditional sub-group. This needs to be clarified throughout the paper and some of the points made in the discussion may need to be reviewed as well.***

The reviewer’s understanding is correct: telephone follow up was only provided for patients not responding electronically. This was stated in the methods (“Non-responders or those without any electronic contacts including those with only home telephone numbers (both pre-operative and post-operative) were contacted by telephone …”) and in the abstract (“Telephone call follow up was used for people that failed to respond electronically”) but has now been reinforced and clarified throughout the manuscript.

The abstract now includes the following sentences:

“Pre-operatively, 73.2% of patients responded electronically and telephone follow-up of non-responders increased this to 91.4%.”

Further, we have changed research question 3 as follows:

“are patient-reported outcomes different between those who provide direct electronic data entry and those who provide information via telephone call after not responding electronically?”

We have also targeted misunderstanding of the results by adding a sentence to the discussion:

“It should be noted that these findings do not allow a direct comparison of primary telephone and electronic follow up, as the telephone follow up was only used for patients who did not or could not respond electronically. Rather, it compares electronic follow up to telephone follow up in people who did not respond electronically.”

***More information is required to understand the size of the various samples that are analysed. For example, p.7 explains why 1,600 patients were dropped from the initial sample of 18,080 patients eligible for the PROMs pilot study but readers are not told why the remaining 4,492 patients were excluded to arrive at a final sample of 11,988 patients.***

We apologise for confusion regarding the flow of numbers. This has been addressed and clarified in the results section. The 18,080 included patients who were later excluded (approximately 5,000 were revisions, not OA, or landline only). A further group had their data externally uploaded from a separate source (another exclusion). However, the original demographic analysis (Tables 1 and 2) included all patients used in the pilot program, whereas analyses including telephone follow up (later tables) was restricted to the time period when telephone follow up were used (this ceased in January 2020). To simplify the paper, we have now restricted ALL analyses to the time period when telephone follow up was used.

The Methods have been edited as follows:

“Between 30 July 2018 and 28 January 2020, the AOANJRR conducted the first stage of a PROMs pilot study, collecting PROMs data from patients pre-operatively and at six months post-surgery from 43 institutions across Australia, including metropolitan and regional, and private and public hospitals from all states and one territory. The analysis includes procedures registered (pre-op analysis) or performed (post-op analysis) between 30 July 2018 and 29 May 2019, to allow 8 months for follow up.”

The results section has been edited as follows:

“Over the study period, 6,224 patients at 43 participating institutions having electronic contact information and primary THA, TKA or TSA for osteoarthritis were registered into the PROMs pilot study.

Of the 6,224 patients registered into the PROMs system, 417 patients had their pre-operative PROMs data collected directly by separate hospital systems, external to the electronic data capture system, and were excluded from pre-op analyses. A further 48 patients were excluded from pre-op analyses due to pre-op follow up phone calls ceasing. For post-op analyses, of the 6,224 patients registered, 24 died and 55 opted out prior to completing their post-op PROMs and were excluded from post-op analyses. A further 573 patients had their procedure after 29 May 2019 and were unable to be followed up by telephone due to post-op follow up phone calls ceasing and are excluded from post-op analyses.

For pre-op analyses, a total of 5,759 patients with a mobile phone and/or email address listed were matched to 6,095 primary hip, knee or shoulder replacement procedures performed for osteoarthritis over the same period and are included in the pre-op analyses. For post-op analyses, a total of 5,572 patients were matched to 5,892 primary hip, knee or shoulder replacement procedures performed for osteoarthritis and are included in the post-op analyses. For patients with multiple procedures, only the first procedure was included in the demographic analyses.”

***It is also unclear why the analysis of post-operative characteristics includes a lower number of patients (3988 + 4211 = 8199). If these discrepancies reflect drop-out then that seems to be much more worrying than the imbalances due to initial non-participation.***

Please see our response to the previous comment. The numbers are now in alignment.

***Finally, Table 2 states that 10,412 patients took part in the pre-operative survey (either electronically or via telephone interview), yet Table 3a reports statistics based on only 1,555 + 399 = 1,984 pre-operative EQ-5D-5L responses. Does this imply that PROMs data were missing for 81.2% of patients participating (i.e. providing some data) in the PROMs pilot study? If so, how meaningful are comparisons based on such a small subsample?***

Table 2 is all patients undergoing hip, knee or shoulder replacement. Tables 3a and 3b (now Tables 4a and 4b) are restricted to patients undergoing total hip replacement. Tables 4a and 4b (now Table 5a and 5b) are restricted to patients undergoing total knee replacement.

Please also see previous response above regarding analysis numbers.

***The discussion should acknowledge that any improvements in representativeness (if that can be shown) as a result of telephone follow-up cannot be attributed to the mode of follow-up but could simply arise from having any follow-up that improves overall participation rates.***

We agree with this point but as the representativeness did not improve and we did not attribute any change to telephone follow up, we feel is it not necessary to suggest other reasons for possible changes.

***The discussion of the cost-effectiveness of telephone follow-up (p. 19) focuses on improvement in representativeness as the sole measure of the ‘value’ of such activities. However, there may also be value in having a larger sample size and, consequently, less statistical uncertainty, for example to construct surgeon-specific performance indicators. The conclusion that the authors reach (“this approach is unlikely to be cost-effective on a large scale if it does not improve representativeness”) is only true under a very narrow definition of value. It also suggests that there may be value in more targeted follow-up with underrepresented patient groups.***

We agree with this point. We have changed the sentence to refer to representativeness as only one aspect and to point out that higher rates of follow up may sometimes be required.

“… this approach is unlikely to be cost-effective on a large scale to improve representativeness but may be used where high rates of follow up are required, e.g., in nested clinical trials.”

***The discussion should make it clear that the findings reported in this paper may not generalise to other clinical registries or jurisdictions.***

This point has been added in the discussion:

“The findings reported in this paper may not generalise to other clinical registries or jurisdictions.”

***p.5 line 75: replace ‘representativeness is biased or is not known’ with ‘sample is unrepresentative’. Sample statistics are not misleading simply because representativeness is not known.***

This has been replaced:

“… whereas an 80% sample may provide misleading information if the sample is unrepresentative”

***P.7 Line 131. Do the authors mean the critical value chosen to reject the alternative hypothesis?***

Yes. This has been changed:

“The critical value chosen to reject the alternative hypothesis was 0.05.”

***P.18 line 210: The authors may wish to consider large-scale PROMs data collections in arthroplasty outside of clinical registries. There have been studies examining non-response patterns in hip and knee replacement in the English PROMs programme (e.g. Hutchings et al 2012 in Health and Quality of Life Outcomes). Similar studies may exist for other national PROMs programmes, such as those in Sweden.***

Thank you. This reference (which concurs with our findings) has been added and one other was found from 2021. Other arthroplasty PROMs reports were found, but without a comparison of responders to non-responders. The discussion has been expanded as follows:

“A previous study in elective surgery patients in England (including hip and knee arthroplasty) showed that responding patients were more likely to be female and older (which concurs with our findings). (11) A recent study patients included in a hip arthroscopy registry also reported that responding patients were more likely to be female and older, but they included a broader age spectrum (more younger people).(12) Similar to our study, the differences between responders and non-responders in both these studies were small. A study of shoulder arthroplasty patients, however, showed that female patients were less likely to respond.(13)”

***p.20 line 244: The authors claim the ‘broad range of demographic […] variables’ as a strength of their study. I would argue that age and sex together do not constitute a broad set of variables. Indeed, I agree with the authors' subsequent statement that other characteristics, such as socioeconomic status and language proficiency but also education and ethnicity are likely to be important. This should be acknowledged as one of the most severe limitations of this study, not a strength.***

The broad range of variables as a strength has been deleted. The subsequent statement has been expanded as a standalone limitation, as follows:

“There may be differences in representativeness for variables not included in this analysis, for example, socioeconomic factors, ethnicity, education and language proficiency.”

**Reviewer #2**

***It was a pleasure to review this manuscript. It is well-written and succinct. The study itself appears to be largely descriptive. I only have a few minor and major comments that will hopefully improve the clarity, accuracy, and impact of this analysis.***

***[Minor] Remove EuroQol from instrument name (EQ-5D-5L is the official name) and "Utility"; should also indicate which country-specific value set was used to calculate the index scores.***

EuroQol has been removed from the Methods. Australian preference weights were used to calculate the index scores. This has been added to the Methods as follows:

“EQ-5D-5L(8) Utility Index (using Australian preference weights)”

***[Minor] Table 1 and 2: add summary of characteristics for overall cohort to better understand whether there are numerical differences compared to the two subgroups with the overall demographics; if for instance, the differences from the responding group are more similar than the non-responding group... it might provide more to the conclusion that non-response may be less of a concern than previously assumed.***

We agree with this point. Columns named “Total” have been added to Tables 1 and 2 containing the demographics for the total population.

***[Minor] Table 3a/b: normative to present EQ-5D index scores and their SEs (or utilities, not "utility score") to 2 or 3 decimal places, depending on the published algorithm.***

We have changed the naming in the tables from “Utility Score” to “Utility Index” as per standard usage. We have changed these to 2 decimal places in the tables.

***[Major] Is race/ethnicity not collected/readily available within this data set? Education level? Marital status? Advance directive status? These demographic characteristics are known to influence PRO measurement. I acknowledge that this is noted in the limitations but seems to be a major limitation that requires further discussion (e.g., which "socioeconomic factors"? How would language proficiency influence PROM measurement?)***

These variables are not available in our dataset. We accept that they may be important and that our study is limited to the data available. We have added the following sentence to the Methods:

“Other demographic information (e.g., education and ethnicity) was not available for analysis.”

***[Major] It occurs to me that there is an opportunity to also report not only the mean PROM scores, but the difference in scores between the pre- and post-operative measurements for a subset of patients who provided both. Given the investigators aim to understand the potential selection bias on PROM as a study endpoint, it seems important to capture the potential bias on changes in the patient experience. Please consider whether this is possible with the current analysis and if not, provide justification.***

Unfortunately, we cannot answer the question about the association between response rate and changes in PROMs because non-responders have not provided PROMs data. We have conducted a separate analysis to answer a separate research question (what is the association between pre-operative PROMs and post-operative response rate?). We feel that adding that analysis to this manuscript would not address the main question of this paper (representativeness) and we aim to publish that analysis separately.

We would be able to publish differences in PROMs between the electronic and telephone responders in this manuscript, but feel that it is not directly related to the research question regarding representativeness based on patient characteristics rather than patient responses.
